# Morphology and Distribution of Primary Carbides in Forged Cr-Ni-Mo-V/Nb Steel

**DOI:** 10.3390/ma17040867

**Published:** 2024-02-13

**Authors:** Yang Li, Tingting Xu, Xin Cao, Zhipeng Wu, Jianwen Fan, Chundong Hu, Han Dong

**Affiliations:** 1 School of Materials Science and Engineering, Shanghai University, Shanghai 200444, China; gody1106040057@163.com (Y.L.); reay777@126.com (Z.W.); fanjw_nwpu@aliyun.com (J.F.); donghan@shu.edu.cn (H.D.); 2 Zhongyuan Special Steel Co., Ltd., Jiyuan 459000, China; xuting_1@163.com; 3 Zhejiang Institute of Advanced Materials, Shanghai University, Jiaxing 314100, China; caoxin961113@163.com

**Keywords:** primary carbide, three-dimensional morphology, non-aqueous solution, electrolytic, distribution characteristics

## Abstract

This study aims to investigate in situ the three-dimensional (3D) morphology and distribution of primary carbides (PCs) in electro-slag remelting (ESR) forged 30Cr_3_Ni_3_Mo_2_V steel. A facile non-aqueous electrolytic etching method was applied to prepare 3D PCs on the matrix. The morphology, composition, and element concentrations of PCs were characterized using a combination of optical microscopy (OM), scanning electron microscopy (SEM) with energy dispersive spectroscopy (EDS), and electron back-scattered diffusion (EBSD). The precipitation, type, and composition of PCs in the same steel were also simulated using Thermo-Calc software Version 2015a. The results indicate that PC is rich in Nb, which is a potential heterogeneous nucleating agent. Both the size and number of PCs increase from the edge to the center of the ingot. The large-sized PCs present three dominant types of morphology, which vary in different regions, i.e., a bulky type dominates in the edge region, a lamellar type dominates in the middle region, and a stripy type dominates in the core region. The results of EBSD analysis show that the orientation of PCs with different morphologies is different and that more nanosized V-rich type carbides are precipitated on the matrix. The thermodynamic calculations show that MC precipitates from the liquid phase when the solid phase fraction is greater than 0.985 and that the MC-type carbides are rich in Nb, which agrees well with the experimental results.

## 1. Introduction

Cr-Ni-Mo-V/Nb steels are widely used in engineering and military materials due to their good hardenability, excellent corrosion resistance, high hardness, and a favorable combination of high strength and high toughness [1,2,3,4,5]. Nevertheless, the existence of comparatively high contents of C, Ni, Mo, and V in Cr-Ni-Mo-V/Nb steels prompts the precipitation of primary carbides (PCs) during the dendritic solidification process. This, in consequence, induces the occurrence of inhomogeneously localized stress within the specific regions where the PCs precipitate. This inhomogeneous stress can easily initiate microcracks near the PCs, which then propagate through the matrix and finally lead to the failure of steels [6,7,8,9]. The morphology and distribution of PCs in the matrix significantly affect the steel performance, i.e., strength, toughness, fatigue life, etc. [10]. Precisely tailoring the morphology and distribution of PCs in a suitable fashion can notably enhance the steel’s performance [11,12,13]. 

The in-situ characterization of the PCs in steels in three dimensions is important, for such characterizations can offer powerful evidence that reveals the precipitation mechanism of carbides, which is vital for the development of control strategies for the improvement of steel performance. Extensive studies have been conducted to explore the morphology and distribution of PCs in steels [14,15,16]. Most of these studies applied a non-aqueous electrolytic extraction method to obtain PC samples from cast ingots, where the matrix is completely dissolved and the PC powders are then collected and purified. The electrolytic extraction method takes a comparatively long time (≥1 h), and, more importantly, the observed PCs no longer exhibit their original appearance and distribution in the matrix.

Recently, a facile non-aqueous electrolytic etching method was adopted by some researchers for in-situ characterization of the three-dimensional (3D) morphology and distribution of carbides in steels. In this method, the steel sample is only partially electrolyzed to expose the 3D carbides for observation purposes, which, thus, only takes several minutes. More importantly, the characterized carbides keep their original appearance and distribution in the matrix. Sun et al. [17] characterized the PCs in a high-carbon martensitic stainless steel by using this partially electrolytic method (which takes 5–7 min) and found that the morphology of the PCs is mainly massive, spherulite, fibrous, and short-rod-shaped at the edge or in the center. In their study, the bulk, fibrous, and short-rod-like carbides are larger, up to 10 μm, while the spherulite carbides are only 2–4 μm in size; the area fraction of the PCs at the edge is 4.62% and that in the center is 5.97%. Huang et al. [18] also used a partially electrolytic method to characterize the PCs in Ce-H13 steel. They observed that the PCs were all located at the grain boundaries and that the morphology of the secondary carbides at different distances from the edge to the sample center showed dendrite structures. They also reported that from the edge to the center, the rapid growth of branch-carbide results in a significant increase in the size of the PCs.

The research on carbides in steels remains crucial for the development of ultrahigh-strength steels. This study aims to investigate the in-situ 3D morphology and distribution of PCs in a forged electro-slag remelting (ESR) 30Cr_3_Ni_3_Mo_2_V steel (medium-carbon medium-alloy type). A facile non-aqueous partially electrolytic method was adopted to expose the PCs from the matrix surface for characterization. Both experiments and simulations were conducted to reveal the morphology, composition, and distribution of PCs in the steel. The results are expected to offer useful guidelines for the development of high-quality medium-carbon medium-alloy steels.

## 2. Materials and Methods

The chemical composition of 30Cr_3_Ni_3_Mo_2_V steel is shown in Table 1. The mass of the ESR ingot was 3000 kg, and the slag system used in the experiment was 65 wt % CaF_2_, 25 wt % Al_2_O_3_, and 10 wt % CaO. The ESR melting rate was 450 kg/h. A sample with a thickness of about 140 mm was obtained at the riser end of the ESR ingot. First, the as-cast structure of the ingot was observed, and it was found that the edge, the 1/2 radius, and the core regions of the ingot had different segregation degrees. Then, the PCs in these three areas were engraved using a non-hydropower method, and the form and distribution of the PCs in each part were characterized by OM, SEM, EBSD, IPP, Thermo-Calc, and other analytical methods. The specific methods used were as follows.

**Figure 1:**
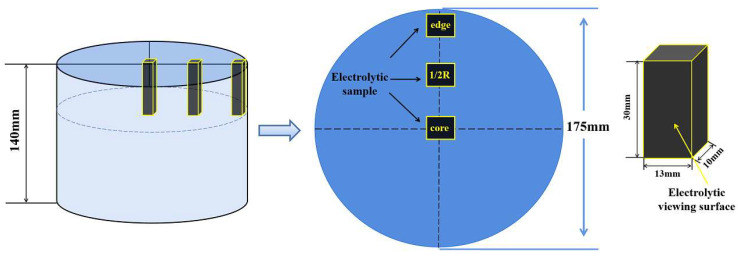
Sampling diagram.

Three cuboid samples with a size of 30 mm × 13 mm × 10 mm were cut from three different locations of the ingot using an electric spark wire cutting machine (Biaole Abrasimet M, Buehler, Lake Bluff, IL, USA), as shown in Figure 1. The surfaces to be characterized on the samples were ground in sequence with 180-, 320-, 600-, 1200-, 1500-, and 2000-mesh SiC sandpapers and then polished with W0.5 diamond polishing paste. The remaining 5 surfaces of the samples were kept free from rust and oil. The samples were cleaned with an ultrasonic cleaner and then air-dried.

Simultaneously, 10 mm × 10 mm × 5 mm samples were taken, and vibratory polishing was performed using 0.02 µm of colloidal silica suspension for microstructural analysis (EBSD) (Bruker QUANTAX EBSD 400i e-FlashFS, Bruker, Billerica, MA, USA).

**Figure 2:**
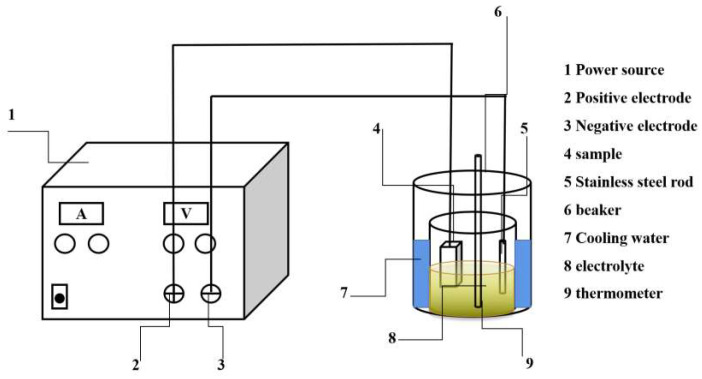
Schematic diagram of the experimental setup for electrolytic etching.

To expose the 3D PCs on the matrix surface, a non-aqueous electrolytic etching method was employed using an experimental setup as illustrated in Figure 2, employing a direct current constant voltage power supply. The specimen was connected to the positive electrode side, while a stainless-steel rod was linked to the negative electrode side. The electrolyte consists of 10% AA solution (i.e., 1 wt % tetramethylammonium chloride + 10 wt % acetylacetone + 89 wt % methanol). In this mixture, methanol acts as the solvent, tetramethylammonium chloride functions as the conductive agent, and acetylacetone serves as the complexing agent. The electrolysis voltage was set at 20 V, with the current density being in the range of 0.10–0.15 A·cm^−2^. The maximum electrolytic etching time was 300 s and the temperature was kept at approximately 0 °C. After the electrolytic etching process, the specimens were rinsed with anhydrous ethanol.

The microstructures of PCs in the samples were characterized using an upright fully automated optical microscope (OM, Carl Zeiss Axio Imager.M2m, Carl Zeiss, Jena, Germany), a field emission scanning electron microscope (SEM, FEI Apreo 2S HiVac, Thermo Fisher Scientific, Waltham, MA, USA), and electron back-scattered diffraction (EBSD) in conjunction with SEM. The composition of the PCs was characterized by energy dispersive spectroscopy (EDS). The number density and area fraction of the PCs were analyzed using Image-Pro Plus 6.0 (IPP) software, combined with SEM and OM.

To explore more deeply the precipitation mechanism of PCs in the steel, three corresponding samples were also taken from a cast ESR ingot, the dendrite microstructures of which were characterized by OM. The cast samples for OM observation were polished first and then subjected to shallow corrosion with nitric acid (4 wt %) and alcohol solution for 5–10 s. The results of the secondary dendrite arm spacing (SDAS) tests were obtained by random counting of the observed secondary dendrite morphology.

The precipitation and composition of PCs of primary carbides and the composition of alloying elements were also calculated using Thermo-Calc software 2015a.

## 3. Results and Discussion

### 3.1. Number and Size Distribution of PCs

**Figure 3:**
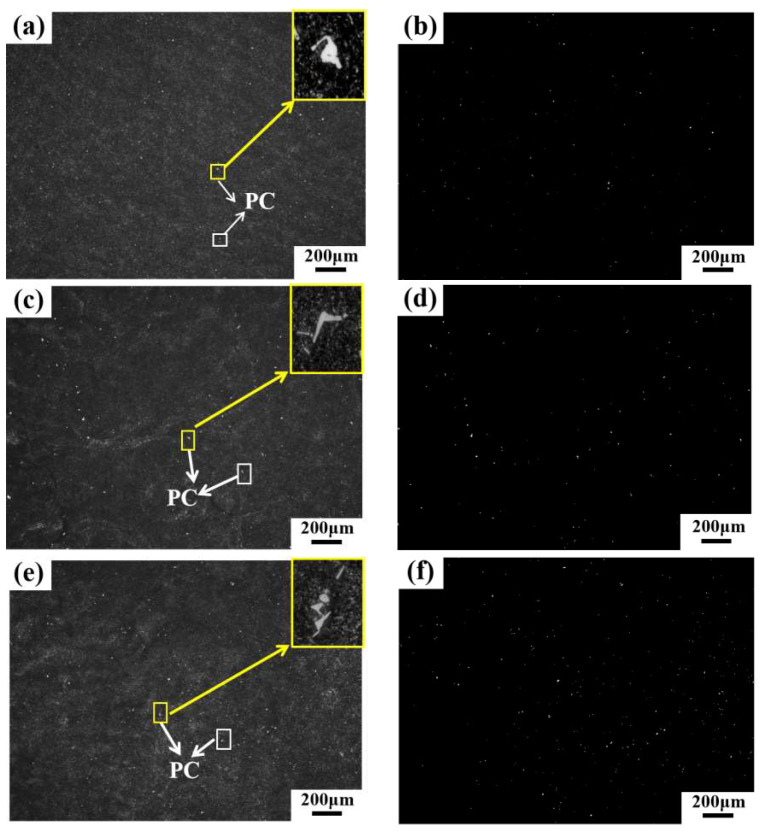
OM images of PCs at three different positions of the ingot: (**a**) the edge region; (**c**) the 1/2 radius region; and (**e**) the core region. Images adjusted by IPP software for PC distribution statistical analysis from the original OM images of: (**b**) from (**a**); (**d**) from (**c**); and (**f**) from (**e**).

The OM images of PCs at three different positions (i.e., the edge, 1/2 radius, and the core) of the ingot are presented in Figure 3a,c,e, respectively. The bright white dots in the images are PCs. With these enlarged views, irregularly shaped PCs were observed.

**Figure 4:**
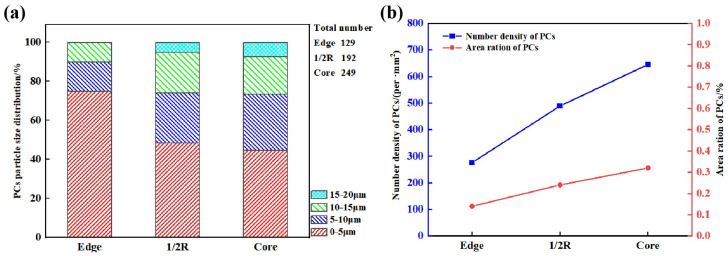
(**a**) Number density, area fraction, and (**b**) size distribution of PCs in three different positions on the ingot, assessed via the IPP software.

To examine in more detail the distribution information of PCs in these regions, statistical analysis using an image processing software (i.e., the IPP) was conducted. Typical images for statistical analysis are shown in Figure 3b,d,f; these were adjusted from the original OM images of Figure 3a,c,e, respectively. Based on such adjusted images, the number density (defined as the particle number per unit area) and area fraction (defined as the ratio between the total projected area of PCs and the total examined region’s area) of PCs can easily be obtained. The number density values of the PCs in Figure 3b,d,f are plotted in Figure 4b, and comprise 276 (the edge region), 489 (the 1/2 radius region), and 645 (the core region) particles/mm^2^, respectively. It can be seen that the number density increases obviously from the edge to the core regions. Thirty images (500× magnification) in each region were used for the assessment of the PC area fractions, the average values of which are also plotted in Figure 4b. The average area fractions of PCs in the three different regions are 0.14%, 0.24%, and 0.32%, respectively, which follows the same trend for the number density from the edge to the core regions.

Thirty randomly selected OM images (at 500× magnification) of the samples from each position (i.e., the edge, the 1/2 radius region, and the core regions) of the ingot were used for analyzing the size distribution of PCs using the IPP software, the results of which are presented in Figure 4b. Based on the 30 images in each position, the obtained total particle numbers of PCs are 129 (the edge region), 192 (the 1/2 radius region), and 249 (the core region), respectively, with the particle number of PCs increasing from the edge to the core regions. This agrees with the particle number density results, as illustrated in Figure 4a.

The observed PC particle size (i.e., the equivalent diameter) ranges between 0 and 20 μm. To better illustrate the particle size distribution (defined as the ratio of the particle number in a specific size zone to the total particle number counted in the examined position of the ingot), the sizes of PCs are divided into four zones: (0, 5), (5, 10), (10, 15), and (15, 20) μm. At the edge region, the sizes of 75.0% of PC particles are in the range of (0, 5) μm, 15% in (5, 10) μm, 10% in (10, 15) μm and 0% in (15, 20) μm. The largest PC particle size at the edge region is measured to be 14.85 μm. At the 1/2 radius region, 48.3% of PC particles have a size in the range of (0, 5) μm, 25.9% in (5, 10) μm, 20.7% in (10, 15) μm and 5.1% in (15, 20) μm. The largest PC particle size at the 1/2 radius region is 16.38 μm. At the core region, only 44.6% of PC particles show a size in the range of (0, 5) μm, 25.9% in (5, 10) μm, 20.7% in (10, 15) μm and 5.1% in (15, 20) μm. The largest PC particle size at the core region is 18.66 μm. It is obvious that both the number and size of PC particles increase from the edge to the core regions.

**Figure 5:**
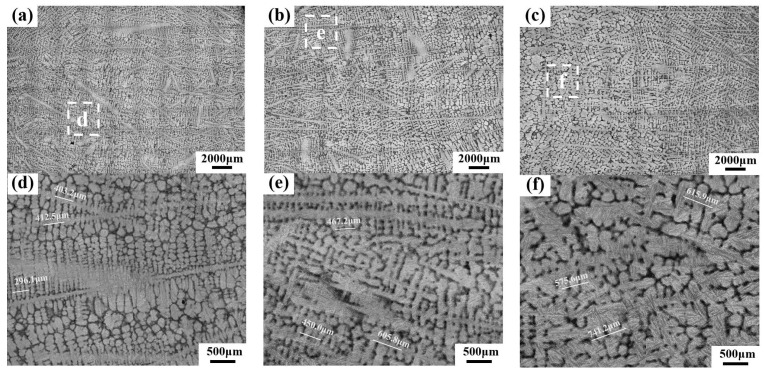
OM images of dendrite microstructures at three different positions in a corresponding cast ingot: (**a**) the edge region; (**b**) the 1/2 radius region; (**c**) the core region. Enlarged images of the white box: (**d**) from (**a**); (**e**) from (**b**); (**f**) from (**c**).

Since PCs predominantly precipitate within the interstices of secondary dendrites, the growth, distribution, and size of PCs are intrinsically linked to the development of the secondary dendrites. To better understand the mechanisms behind the number and size distribution of PC particles in the forged ingot, the microstructures of secondary dendrites in the corresponding regions of a cast ingot were also characterized, the results of which are shown in Figure 5. Figure 5a–c shows the dendrite morphology of the edge, the 1/2 radius, and the core regions of the ingot, respectively; Figure 5d–f shows the enlarged images of the white boxes in Figure 5a–c, respectively. The SDAS (measurement methods refer to method B in the literature [19]) at the three positions of the ingot were 123.4 μm, 169.1 μm, and 281.0 μm, respectively. It is evident that from the edge to the core regions, the secondary dendrites coarsen, with the interstices between them becoming larger. This explains why the size of the PC particles increases from the edge to the core regions.

The edge region, which is closest to the crystallized wall, has the fastest cooling rate. This gives rise to the development of finely crystalline domains that are characterized by narrow inter-dendritic spaces, which, in turn, confine the sizes of the PC particles that precipitate within these dendritic interstices. In addition, the fast cooling rate may foster the creation of dendritic closed zones, which stops the further flow of the residual molten steel within the closed zones and thereby impedes the accumulation of carbon and alloying elements. Furthermore, as the cooling rate accelerates, the overall solidification process is expedited, resulting in a reduction in the nucleation of PC particles [5,15].

### 3.2. Morphology and Distribution of PCs

**Figure 6:**
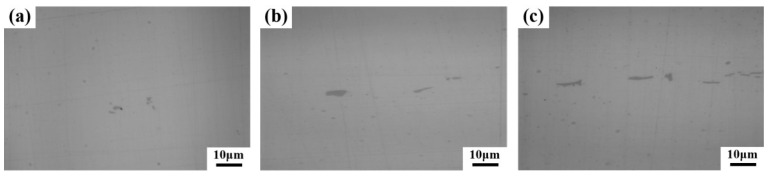

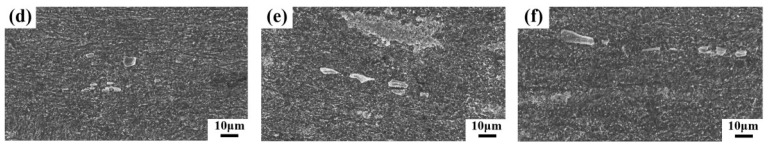
OM images of the 2D morphology of PCs at three different positions on the forged ingot: (**a**) the edge region; (**b**) the 1/2 radius region; (**c**) the core region. SEM images of the 3D morphology of PCs at three different positions on the forged ingot: (**d**) the edge region; (**e**) the 1/2 radius region; (**f**) the core region.

The two-dimensional (2D) morphology of the PCs was characterized by OM using the polished non-etching samples, while the three-dimensional (3D) morphology of PCs was characterized by SEM using the 300-second-etching samples. Representative 2D and 3D morphology of the PCs at the three different positions (i.e., the edge, 1/2 radius, and core regions) of the forged ingot was presented in Figure 6. In the OM images, the dark grey represents PC particles, while the light grey indicates the matrix. In the SEM images, the bright gray represents PC particles.

As shown in Figure 6a,d for the edge region, several discrete, comparatively large (0–5 μm), irregular blocky PCs were observed, with multiple and much smaller PCs dispersed through the matrix. In the 1/2 radius region (Figure 6b,e), the aspect ratios of the comparatively large PCs become larger. This type of PC is referred to as a lamellar type. The size of the lamellar also PCs becomes larger (5–10 μm) compared to the irregular blocky PCs in the edge region. In the core region (Figure 6c,f), the typical stripy morphology for the comparatively large PCs was observed, with the stripy PCs showing the same orientation direction and tending to connect in the stripe direction. The size of the PCs in the core region is the largest (10–15 μm).

**Figure 7:**
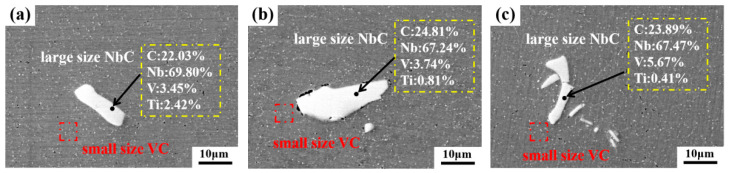
SEM images with EDS analysis for 2D PCs at three different positions on the forged ingot: (**a**) the edge region; (**b**) the 1/2 radius region; (**c**) the core region.

**Figure 8:**
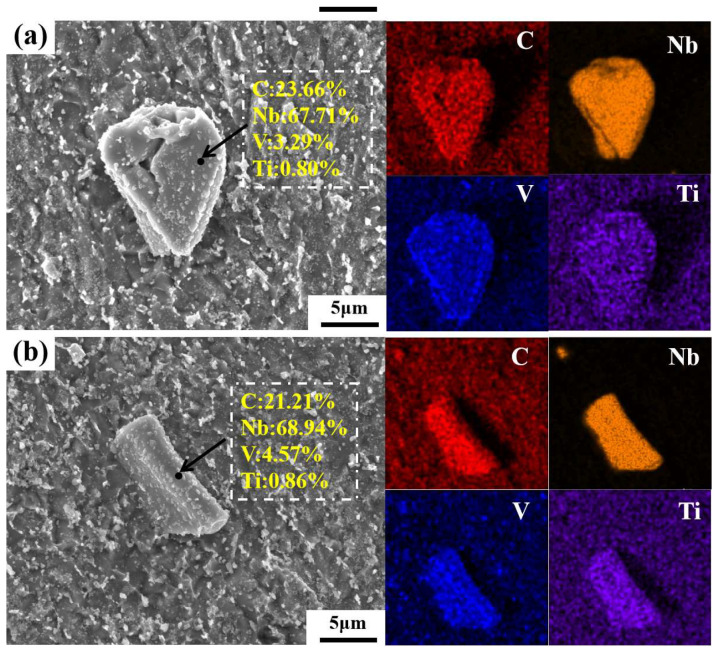

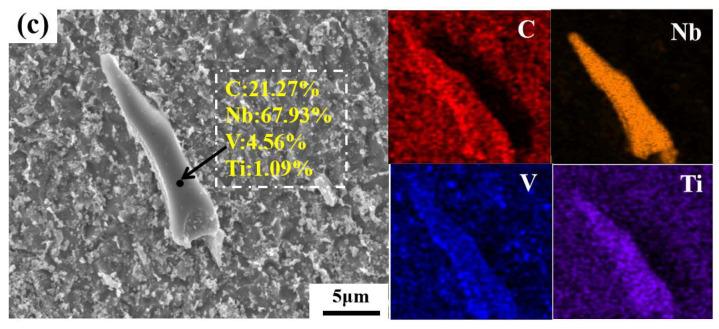
SEM images with EDS maps for three typical types of 3D PCs from three different positions on the forged ingot: (**a**) the blocky type in the edge region; (**b**) the lamellar type in the 1/2 radius region; (**c**) the stripy type in the core region.

The composition of the three typical types of PCs in the three different positions (i.e., the edge, 1/2 radius, and core regions) on the forged ingot was examined via SEM with EDS, the results of which are shown in Figure 7 and Figure 8. Figure 7 shows the SEM images along with EDS analysis for the morphology and composition of the 2D PCs in the polished non-etched samples. Figure 8 presents the SEM images along with EDS maps for the morphology and composition of the 3D PCs in 300-second-etching samples. In both Figure 7 and Figure 8, the EDS results indicate that all three types of large-sized PCs at the three different positions show a similar composition, with the presence of Nb (67.24–69.80%), V (3.29–5.67%) and T (0.41–2.42%) elements indicating the Nb-rich type of PCs. In Figure 7, multiple nanosized V-rich type carbide particles, uniformly distributed through the matrix, were clearly observed.

**Figure 9:**
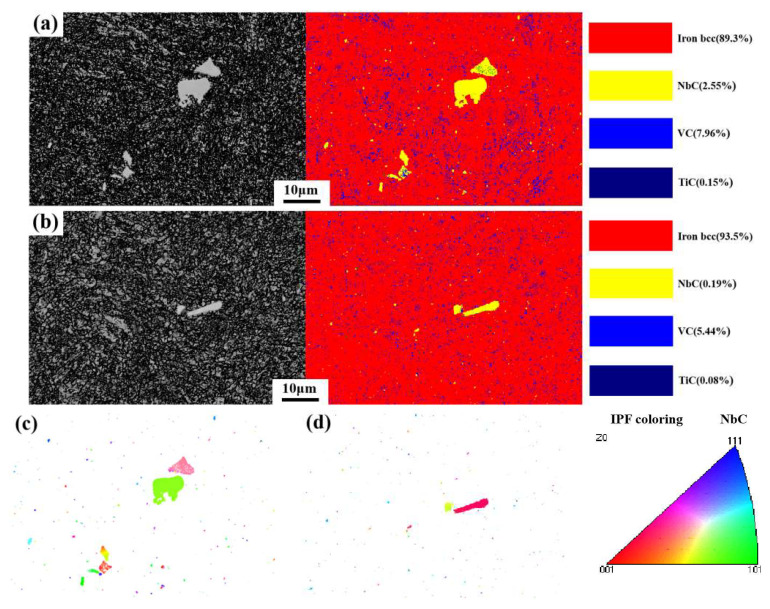
EBSD phase and orientation maps for typical 2D (**a**,**c**) irregular blocky and (**b**,**d**) stripy PCs.

To explore more deeply the distribution, composition, and growth orientation of PCs, the EBSD phase and corresponding orientation maps for two typical irregular blocky and stripy 2D PCs were characterized, based on the non-etched samples from the edge and core regions of the forged ingot. The results are shown in Figure 9. The yellow regions denote Nb-rich carbides, the blue regions signify V-rich carbides, and the red regions represent body-centered cubic (BCC) ferrite. The results in Figure 9a,b further confirm that the large-sized PCs in both the edge and core regions of the ingot are Nb-rich. These results also indicate that the nanosized carbides dispersed through the matrix are V-rich. The observed distribution of the nanosized V-rich type carbides agrees well with the phenomenon observed in Figure 7. Figure 9c,d, respectively, shows the orientation distribution of large-sized Nb-rich PCs, showing the different orientations of these two carbides.

**Figure 10:**
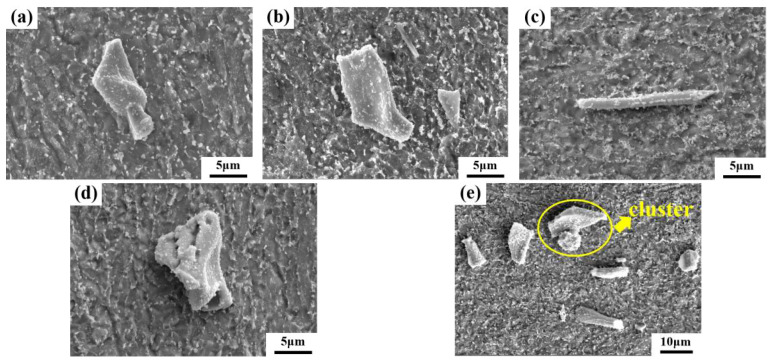
SEM images of 3D morphology of PCs in the forged ingot: (**a**) typical blocky type at the edge region; (**b**) typical lamellar type at the 1/2 radius region; (**c**) typical striped type at the core region; (**d**) skeleton-like blocky PC at the edge region; (**e**) PC cluster at the core region.

To better statistically analyze the distribution of PCs in the forged ingot, PCs were categorized into three types, based on their morphological characteristics: (1) the blocky type, with both aspect and flakiness ratios of less than 3 (a typical example is Figure 10a); (2) the lamellar type, with an aspect ratio of less than 3 and a flakiness ratio of greater than or equal to 3 (a typical example is Figure 10b); (3) the stripy type, with both the aspect and flakiness ratios greater than or equal to 3 (a typical example is Figure 10c). Besides the typical morphology, some PCs exhibit peculiar shapes, such as the skeleton-like blocky shape shown in Figure 10d, which was observed at the edge region. A small number of PC clusters were also observed at the core region, as shown in Figure 10e. These clusters are generally formed by a connection of 2 or 3 individual PC particles.

**Figure 11:**
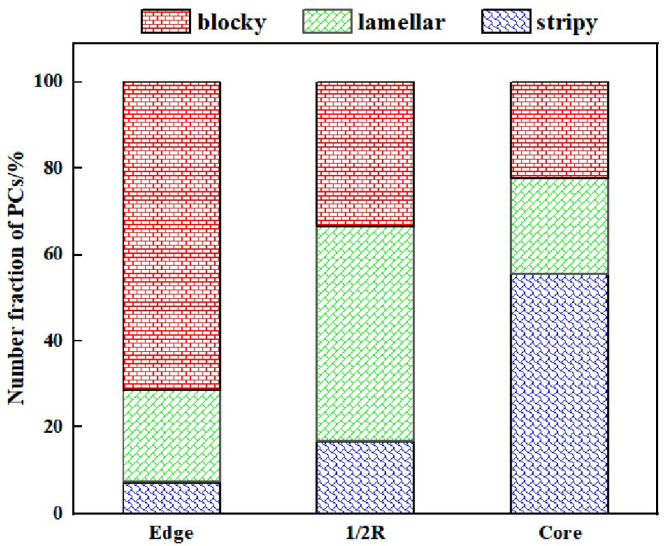
Number distribution of large-sized 3D PCs at different positions of the forged ingot.

Twenty representative SEM images (10,000× magnification) from each position were used for statistical analysis of the distribution of the large-sized 3D PCs in the forged ingot, the results of which are shown in Figure 11. The *Y*-axis represents the number fraction of a specific shape of PC, which is calculated by the ratio of the number of the specifically shaped PCs to the total number of all shaped PCs. At the edge region, 71.43% of PCs exhibit a blocky morphology, while 21.43% are of the lamellar type and 7.14% are of the stripy type. At the 1/2 radius region, the predominant PC morphology is of a lamellar shape (50.00%), followed by the blocky shape (33.34%), and then the stripy shape (16.67%). At the core region, stripy PCs dominate (55.56%), with the blocky and lamellar PCs having similar proportions (~22.22%).

At the edge of the ESR ingot, which is characterized by a high cooling rate and minimal secondary dendrite spacing, primary carbides experience limited time and space for nucleation and growth within the gaps between secondary dendrites. Consequently, they exhibit predominantly blocky shapes and smaller sizes. Moving to the 1/2 radius, the reduced cooling rate and increased secondary dendrite spacing afford the carbides more time and space to develop within the dendrite gaps. This leads to a gradual increase in their sizes and a reduction in the number of small-sized carbides, resulting in the formation of lamellar and blocky carbides. In the center, where the cooling rate is the slowest and the secondary dendrite spacing is the largest, carbides have ample time and space for complete nucleation and growth within the dendrite gaps. The solidification process approaches equilibrium, giving rise to larger-sized carbides, a higher number of carbides, and a propensity to form stripy carbides.

### 3.3. Thermodynamic Calculations

**Figure 12:**
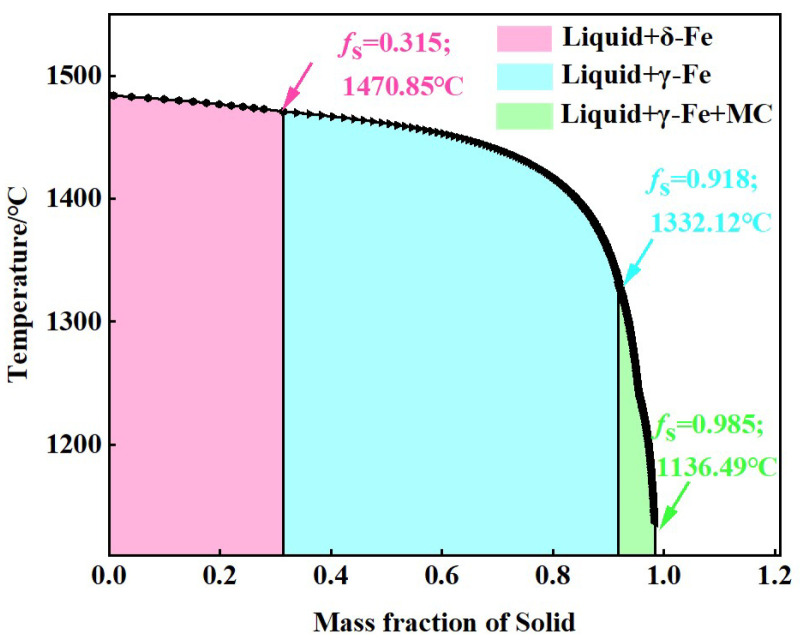
Precipitation order of phases in the non-equilibrium solidification of 30Cr_3_Ni_3_Mo_2_V steel.

To better understand the precipitation of PCs in 30Cr_3_Ni_3_Mo_2_V steel, a Scheil model created in Thermo-Calc was used to simulate the phases during the solidification process of 30Cr_3_Ni_3_Mo_2_V steel, the result of which is shown in Figure 12.

At a temperature of 1484.1 °C, high-temperature ferrite first precipitates from the liquid phase. When the temperature decreases to 1470.9 °C and there is a solid fraction of 0.315, austenite begins to precipitate from the liquid phase. During the solidification process, the variation in the solidification rate leads to a lower Cr/Ni ratio at the base metal and fusion zone. At the same time, a higher level of Cr/Ni at the heat-affected zone (HAZ) indicates that the segregation rate of HAZ ferrite is higher, indicating that the high-temperature ferrite precipitates before austenite [20]. As the temperature continues to decrease to 1332.1 °C and there is a solid fraction of 0.918, an eutectic reaction occurs in the residual liquid phase, and MC-type PCs begin to precipitate. At this point, the phase composition consists of austenite, MC-type PCs, and the residual liquid phase. When the temperature reaches 1136.5 °C, the solid fraction approaches 0.985, which can be considered to indicate that the molten steel has completely solidified. According to the thermodynamic calculations, MC-type PCs precipitate at the end period of the solidification of 30Cr_3_Ni_3_Mo_2_V steel.

**Figure 13:**
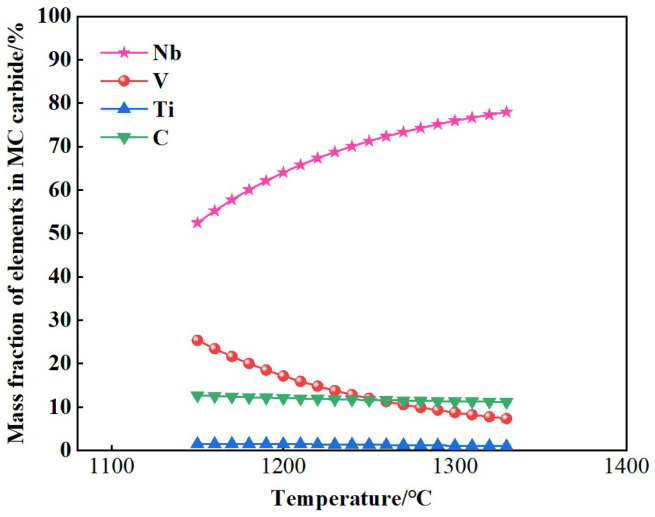
Alloying element concentrations in MC-type PCs.

The element concentrations of Nb, V, Ti, and C in MC-type PCs during the non-equilibrium solidification process of 30Cr_3_Ni_3_Mo_2_V steel were also simulated, the results of which are presented in Figure 13. These results indicate that the MC-type PCs are rich in Nb, which is consistent with the experimental results shown in Figure 7, Figure 8 and Figure 9.

## 4. Conclusions

A facile non-aqueous electrolytic etching method was applied to prepare PC samples for the in-situ characterization of the morphology and distribution of 3D PCs in forged 30Cr_3_Ni_3_Mo_2_V steel. The conclusions are as follows.

The ESR forged ingot of 30Cr_3_Ni_3_Mo_2_V steel contained micro-sized large PCs ((0, 20) μm, hundred particles/mm^2^), with multiple nanosized small carbides dispersed through the matrix. Both the average size and number density of the large PCs increased from the edges to the core regions of the ingot. The average size mainly increased from (0, 5) μm to (10, 20) μm, and the number density increased from 276 particles/mm^2^ to 645 particles/mm^2^.

The large-sized PCs showed three typical morphologies, i.e., the blocky, lamellar, and stripy types. The blocky type of PCs (71.43% number fraction) was predominant in the edge region of the ingot, while the lamellar type (50.00% number fraction) dominated in the 1/2 radius region, and the stripy type (55.56% number fraction) is the most prevalent in the core region. The orientation of PCs with different morphologies was different.

The large-sized PCs were rich in Nb (67.24–69.80%), with minor V (3.29–5.67%) and minor Ti (0.41–2.42%). The multiple nanosized carbides were V-rich.

The type and composition of PCs in 30Cr_3_Ni_3_Mo_2_V steel as simulated using Thermo-Calc software agree well with the experimental results. The thermodynamic calculations indicated that MC-type PCs precipitate from the liquid phase when the solid fraction (*f**_S_***) exceeds 0.985.

## Figures and Tables

**Table 1:** Chemical composition of the forged ingot (mass fraction, %).

C	Si	Mn	Cr	Ni	Mo	V	Nb	Ti
0.30	0.25	0.36	2.96	3.50	2.00	0.80	0.097	0.004

## Data Availability

Data are contained within the article.

## References

[1] Du Y.F., Lu H.H., Shen X.Q. (2022). Coupled effects of banded structure and carbide precipitation on mechanical performance of Cr–Ni–Mo–V steel. Mater. Sci. Eng. A.

[2] Jo M.G., Ryu S.H., Kim K.I., Kim D.E., Kim J.I., Kim K.T., Kim S.S., Cho G.S. (2022). A Study on the Microstructures and Mechanical Properties of Ni-Cr-Mo-V Low Alloy Steels. Korean J. Met. Mater..

[3] Ziza A., Mikhailov M., Tsukanov V., Nikolaev D., Lychagina T. (2018). A study of retained austenite transformation during high-strength Cr-Ni-Mo-V steel tempering. Lett. Mater..

[4] Jahangiri M., Aieneravaie M., Bayani H., Mehdizadeh M. (2024). Development of an experimental-analytical method for obtaining optimal two-layer welding window of a Ni–Cr–Mo–V alloy steel. Int. J. Press. Vessel. Pip..

[5] Chotěborský R. (2013). Effect of heat treatment on the microstructure, hardness and abrasive wear resistance of high chromium hardfacing. Res. Agric. Eng..

[6] Mishnaevsky L.L., Lippmann N., Schmauder S. (2022). Micromechanisms and modelling of crack initiation and growth in tool steels: Role of primary carbides. Int. J. Mater. Res..

[7] Janovec J., Svoboda M., Výrostková A., Kroupa A. (2005). Time–temperature–precipitation diagrams of carbide evolution in low alloy steels. Mater. Sci. Eng. A.

[8] Hamidzadeh M.A., Meratian M., Saatchi A. (2013). Effect of cerium and lanthanum on the microstructure and mechanical properties of AISI D2 tool steel. Mater. Sci. Eng. A.

[9] Mao M.T., Guo H., Wang F., Sun X. (2019). Effect of cooling rate on the solidification microstructure and characteristics of primary carbides in H13 steel. ISIJ Int..

[10] Li J., Li J., Wang L.L., Zhu Q.T. (2016). Study of the Effect of Trace Mg Additions on Carbides in Die Steel H13. Met. Sci. Heat Treat..

[11] Zheng C., Li L., Yang W., Sun Z. (2014). Microstructure evolution and mechanical properties of eutectoid steel with ultrafine or fine (ferrite + cementite) structure. Mater. Sci. Eng. A.

[12] Liu H., Fu P., Liu H., Sun C., Gao J., Li D. (2017). Carbides evolution and tensile property of 4Cr5MoSiV1 die steel with rare earth addition. Metals.

[13] Kim K.H., Park S.D., Kim J.H., Bae C.M. (2012). Role of spheroidized carbides on the fatigue life of bearing steel. Met. Mater. Int..

[14] Zhu Q.T., Li J., Zhang J., Shi C.B., Li J.H., Huang J. (2019). Precipitation mechanism and reduction of amount of primary carbides during electroslag remelting of 8Cr13MoV stainless steel. Metall. Mater. Trans. B.

[15] Qi Y.F., Li J., Shi C.B., Zhang Y., Zhu Q.T., Wang H. (2017). Effect of directional solidification of electroslag remelting on the microstructure and primary carbides in an austenitic hot-work die steel. J. Mater. Process. Technol..

[16] Mao M.T., Guo H.J., Wang F., Sun X.L. (2019). Chemical composition and structural identification of primary carbides in as-cast H13 steel. Int. J. Miner. Metall. Mater..

[17] Sun C., Li J., Zhang J., Yan W., Li S.H. (2023). Formation and evolution of primary carbides in high-carbon martensitic stainless steel. J. Iron Steel Res. Int..

[18] Huang Y., Cheng G., Li S., Dai W. (2020). Distribution characteristics and thermal stability of primary carbide in cast Ce-H13 steel. ISIJ Int..

[19] Vandersluis E., Ravindran C. (2017). Comparison of measurement methods for secondary dendrite arm spacing. Metallogr. Microstruct. Anal..

[20] Kumar S.P., Chakkravarthy V., Mahalingam A., Rajeshshyam R., Sriraman N., Marimuthu P., Narayan R.L., Babu P.D. (2023). Investigation of crystallographic orientation and mechanical behaviour in laser-welded stainless steel 316L additive components. Trans. Indian Inst. Met..

